# Integrative Circuit-Host Modeling of a Genetic Switch in Varying Environments

**DOI:** 10.1038/s41598-020-64921-5

**Published:** 2020-05-20

**Authors:** Jordan J. Sickle, Congjian Ni, Daniel Shen, Zewei Wang, Matthew Jin, Ting Lu

**Affiliations:** 1 Center for Biophysics and Quantitative Biology, University of Illinois at Urbana-Champaign, Urbana, IL 61801 USA; 2 Center for Advanced Bioenergy and Bioproducts Innovation, University of Illinois at Urbana-Champaign, Urbana, IL 61801 USA; 3 Dougherty Valley High School, San Ramon, CA 94582 USA; 4 Monte Vista High School, Danville, CA 94506 USA; 5 University Laboratory High School, Urbana, IL 61801 USA; 6 Department of Bioengineering, University of Illinois at Urbana-Champaign, Urbana, IL 61801 USA; 7 Department of Physics, University of Illinois at Urbana-Champaign, Urbana, IL 61801 USA; 8 Carl R. Woese Institute for Genomic Biology, University of Illinois at Urbana-Champaign, Urbana, IL 61801 USA

**Keywords:** Differential equations, Genetic circuit engineering, Synthetic biology

## Abstract

Synthetic biology is advancing into a new phase where real-world applications are emphasized. There is hence an urgent need for mathematical modeling that can quantitatively describe the behaviors of genetic devices in natural, fluctuating environments. We utilize an integrative circuit-host modeling framework to examine the dynamics of a genetic switch and its host cell in varying environments. For both steady-state and transient cases, we find increasing nutrient reduces the bistability region of the phase space and eventually drives the switch from bistability to monostability. In response, cellular growth and proteome partitioning experience the same transition. Antibiotic perturbations cause the similar circuit and host responses as nutrient variations. However, one difference is the trend of growth rate, which augments with nutrient but declines with antibiotic levels. The framework provides a mechanistic scheme to account for both the dynamic and static characteristics of the circuit-host system upon environmental perturbations, underscoring the intimacy of gene circuits and their hosts and elucidating the complexity of circuit behaviors arising from environmental variations.

## Introduction

Research spanning two decades has demonstrated synthetic gene circuits as valuable tools for a wide variety of novel applications^1–3^. For example, they have been utilized for directing spatial patterns^4,5^, regulating chemical biosynthesis^6^, generating temporal dynamics^7–9^ and establishing defined ecologies^10–13^. To date, most of the circuits are demonstrated in laboratory conditions where environmental variables like temperature, pH and nutrient level are well-controlled. Such simplified settings have been critical for the design, construction and test of proof-of-concept synthetic devices.

**Figure 1:**
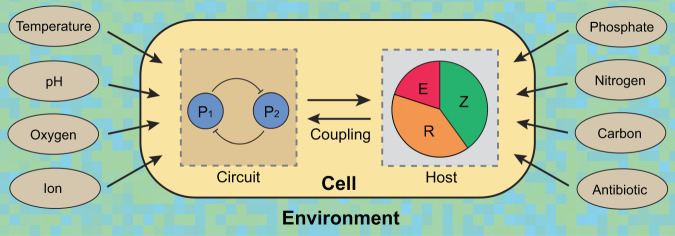
Conceptual illustration of gene circuits and their host in complex environments. In natural settings, cells are subjected to a variety of perturbations and undergo dynamic resource allocation between the circuit and the host in response to environmental variations.

From concept demonstrations to applications, one ultimate goal of synthetic biology is to deploy engineered gene circuits to fulfill tasks in real world^14^. To that end, gene circuits must be able to operate and generate functions in natural complex settings—such as soil, waste water and the human gastrointestinal tract—which fluctuate constantly over time and space. As the physiology of a cell relies on its habitat, environmental fluctuations naturally cause the variations of cellular physiological states. Additionally, gene circuits utilize the building blocks, energy and machinery of their host cells to function; thus, environmental variations that influence the host also shape the behaviors of the circuits consequently (Fig. 1).

Mathematical modeling has emerged as an important methodology for guiding the rational design of genetic circuits^15,16^. To facilitate field applications, these models need to have a capacity of quantitative and predictive descriptions of circuit dynamics in complex environments. Currently, most models consider synthetic circuits orthogonal to their hosts, which has yielded many valuable insights for circuit dynamics. However, a growing pool of evidence shows that circuits and hosts are inherently coupled, and their coupling can significantly alter circuit function^17–20^. For example, an auto-activating circuit that is based on T7 RNA polymerase can significantly lower the growth rate of its host, which in turn leads to the emergent circuit bistability^21^. Recently, integrative modeling, which fully acknowledges circuit-host interactions, has been proposed as a novel scheme to model gene circuit behaviors^22,23^. This coarse-grained, dynamic modeling approach explicitly includes multi-layered interactions between circuits and their host caused by crosstalk and resource sharing. Importantly, the approach involves a mechanistic description of host physiology including cellular growth, translation, and proteome partitioning which are intrinsically subject to environments. Therefore, the framework has the potential to capture environmental regulation of gene circuit behaviors (Fig. 1).

In this study, we explore the potential of the integrative modeling for capturing the behaviors of gene circuits in complex environments. Specifically, using the genetic toggle switch^7^ as our model circuit and *E. coli* as our cellular host, we examine both the steady-state behaviors and transient dynamics of the circuit and the host upon nutrient and antibiotic variations. Our results elucidate complex environmental dependence of circuit behaviors and showcase the power of the integrative framework for understanding circuits under complex settings.

## Results

### Steady-state switch and host behaviors at various nutrient levels

We began our exploration by investigating the steady-state behaviors of the switch and its host under the alteration of nutrient availability. Specifically, we leveraged an integrative circuit-host modeling framework we recently developed^22^, which contains three fundamental parts including a coarse-grained host physiology description, a detailed kinetic module of synthetic circuits and layered circuit-host coupling. The host part utilizes the bacterium *E. coli* as an example and focuses on carbon fluxes from extracellular nutrient to cellular biomass using a coarse-grained approach^24–26^. The underlying processes include carbon uptake, ATP generation, amino acid synthesis, transcription and translation. The associated molecular species are carbon sources, ATP, amino acids, RNAs and proteins. Importantly, RNAs are binned based on their functions into three sectors, including mRNAs encoding proteins, tRNAs delivering amino acids to ribosomes, and ribosomal RNAs (rRNA) that are involved in ribosomes. Proteins and associated mRNAs are classified into three categories including ribosomal and affiliated proteins (R sector), metabolic proteins (E sector) and other proteins (Z sector). Additionally, the cell has an ability to adjust its proteome upon amino acid shortage, which is described by a kinetic description of the alarmone ppGpp^27,28^. The circuit part corresponds to biomolecules generated by synthetic gene circuits. In this study, the genetic toggle switch is the circuit of interest, which brings an additional sector, H, for both proteins and RNAs from gene circuits. The circuit-host coupling includes elementary and ppGpp-induced host-to-circuit interactions as well as load- and functionality-associated circuit-to-host interactions. In this study, we consider only the elementary host-to-circuit interaction and load-associated circuit-to-host interaction.

Using the integrative model, we first examined how nutrient alters the phase diagram of the circuit by simulating the circuit-host system and comparing its steady states under different nutrient levels ([*n*]) (Supplementary Information, Section 2). The values of the model parameters, except the induction strengths of Protein 1 and Protein 2 of the switch (*k*_1_ and *k*_2_ respectively), are all listed in Tables S2 and S3, with the former containing parameters adopted from the previous model^22^ and the latter containing those introduced in this study. For each nutrient level, the phase diagram was numerically drawn in the *k*_1_ − *k*_2_ space by comparing the circuit’s steady states from the simulations starting with two initial conditions. Initial condition 1 (I.C. 1) corresponds to a high level of Protein 1 ([*P*_1_]), a high level of mRNA 1, a low level of *P*_2_ ([*P*_2_]), and a low level of mRNA 2 while initial condition 2 (I.C. 2) is the opposite (Supplementary Information, Section 2). The system is defined bistable if the steady states from the two initial conditions do not converge and monostable otherwise.

**Figure 2:**
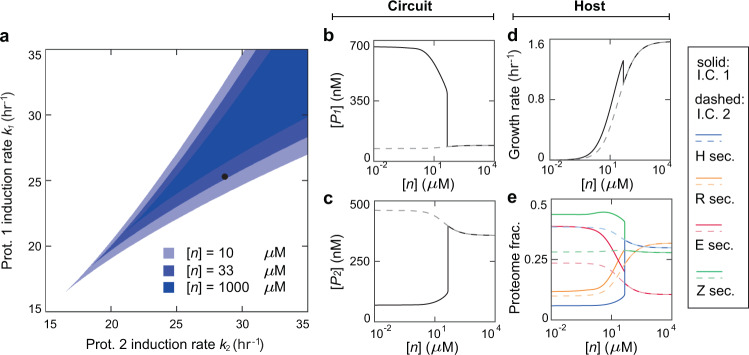
Steady-state circuit and host behaviors upon nutrient variations. (**a**) Bistability diagrams at the nutrient of 10, 33 and 1000 *μ*M. The blue beak areas are bistability regions with darker colors corresponding to higher nutrient levels. The black dot (*k*_1_ = 25 *hr*^−1^, *k*_1_ = 28 *hr*^−1^) is the specific condition under which the circuit-host system is further explored in Panels b–e. (**b**,**c**) Steady-state concentrations of Protein 1 ([*P*_1_]) (**b**) and Protein 2 ([*P*_2_]) (**c**) as a function of nutrient. The protein level is bistable when the nutrient concentration is below 42 *μ*M and monostable otherwise. (**d**) Steady-state growth rate of the host as a function of nutrient. The growth exhibits a similar bistatility-to-monostability transition as circuit prot**e**ins. (**e**) Proteome mass fractions as functions of nutrient. Blue, orange, red and green lines are heterologous proteins from the circuit (H sector), ribosomal proteins (R sector), enzymes (E sector), and remaining proteins (Z sector) respectively. For Panels b–e, solid and dash lines correspond to the steady states evolved from the initial condition 1 (I.C. 1) and the initial condition 2 (I.C. 2) respectively.

Figure 2a shows the overlaid phase diagrams for the nutrient levels of 10 (light blue), 33 (blue) and 1000 (dark blue) *μ*M. We found that increasing the nutrient level shifts the bistability diagram to the upper right, which is consistent with previous reports^29^. The reason is following: nutrient augment increases the host’s growth rate, which enhances the dilution of the proteins; as a result, the circuit production needs to be induced at a higher level to overcome the growth-induced protein dilution for the switch to maintain stable steady states. For the same reason, for a given induction of *k*_1_ and *k*_2_ (e.g., the dot in Fig. 2a at *k*_1_ = 25 hr^−1^ and *k*_2_ = 28 hr^−1^), the circuit, which is originally bistable at a low nutrient, can become monostable at a high nutrient.

In addition to phase diagrams, we investigated the responses of specific key circuit and host variables upon nutrient variations. Figure 2b,c shows the level of circuit proteins (Proteins 1 and 2) as a function of nutrient for a given set of production induction levels (*k*_1_ = 25 hr^−1^ and *k*_2_ = 28 hr^−1^). Notably, the levels of both proteins have two stable states at a low level of nutrient, while beyond the critical level (45.4 *μ*M), the circuit becomes monostable. Correspondingly, host variables also show the same bistability-to-monostability transition. Figure 2d,e illustrates the growth rate of the host upon nutrient alteration and the corresponding partition of cellular proteome, suggesting a strong correlation between the circuit and the host. Interestingly, in the bistability regime, the steady state with a high Protein 1 concentration ([*P*_1_]) (solid line, Fig. 2b) and low Protein 2 concentration ([*P*_2_]) (solid line, Fig. 2c) corresponds to a higher growth rate (solid line, Fig. 2d); in contrast, the low [*P*_1_] (dashed line, Fig. 2b) and high [*P*_2_] (dashed line, Fig. 2c) corresponds to a lower growth rate (dashed line, Fig. 2d). This observation is owed to the larger metabolic load of Protein 2 than that of Protein 1. Associated with the growth rate, proteome allocation (Fig. 2e) exhibits a similar correspondence where, in the steady state of low [*P*_1_] and high [*P*_2_], the host has a greater heterologous H sector (blue lines) but reduced R (orange lines), E (red lines), and Z (green lines) sectors.

**Figure 3:**
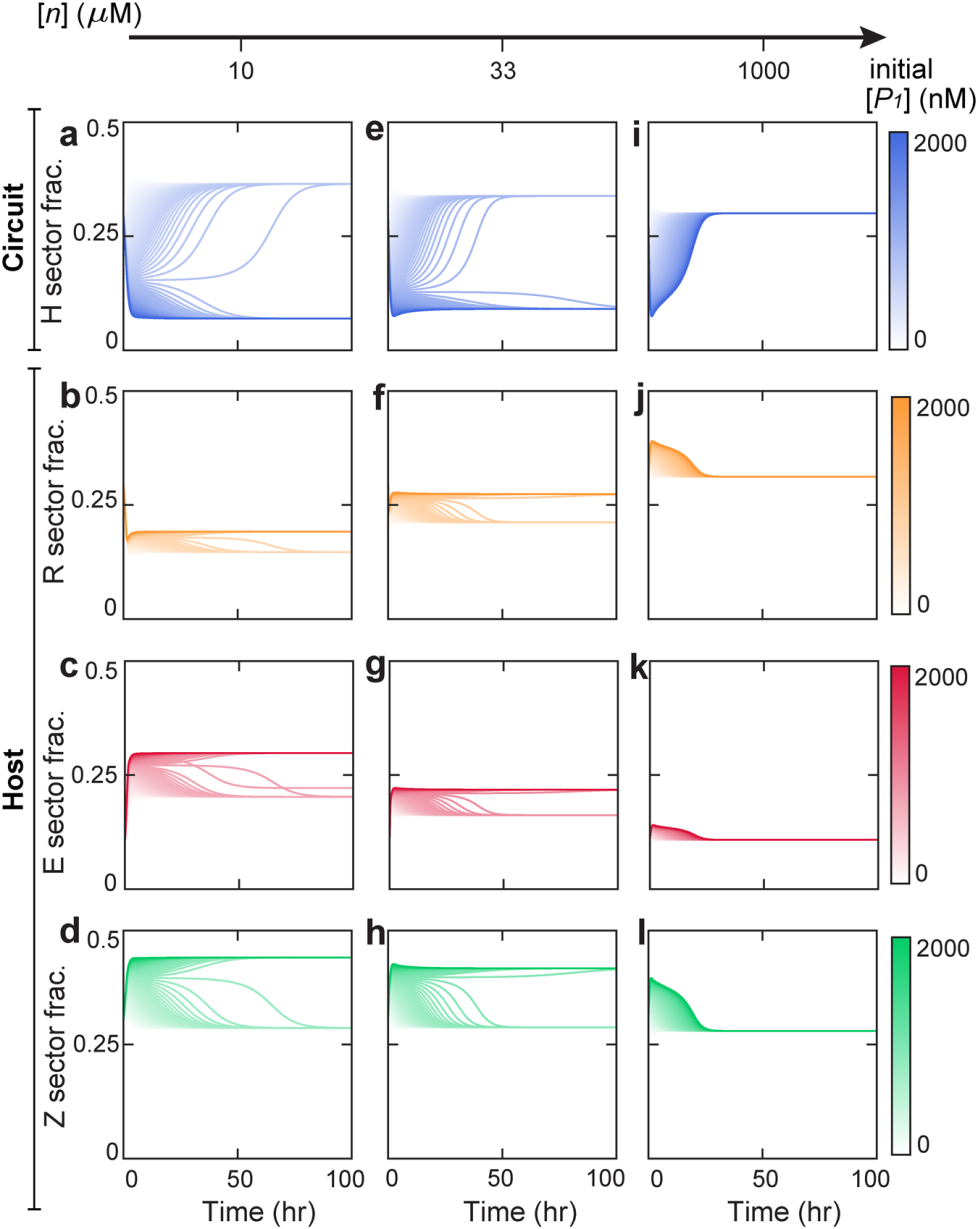
Temporal evolution of cellular proteomes at different nutrient levels. The left, middle and right columns correspond to the nutrient of 10, 33, and 1000 *μ*M accordingly. For each panel, the colors of the trajectories from light to dark correspond to altered the initial concentration of Protein 1 ([*P*_1_]) from 0 to 2000 nM. Other initial conditions remain invariant. Parameters used here correspond to the dot in Fig. 2a.

To intuitively illustrate how nutrient level alters circuit behaviors, we simulated the temporal evolution of key circuit and host variables for different initial conditions and varied nutrient levels. To implement our simulations, the initial [*P*_1_] was varied from 0 to 2000 nM while the initial values of other variables were kept invariant. Figure 3 shows the transient behaviors of the H, R, E, and Z sectors (top to bottom rows) of cellular proteome at the nutrient levels of 10 (left column), 33 (middle column) and 1000 *μ*M (right column). Accordingly, Fig. S1 shows the behaviors of the corresponding [*P*_1_], [*P*_2_] and growth rate at each of these nutrient levels. Notably, the circuit and host variables diverge to two distinct stable states for different initial conditions at the nutrient levels of 10 *μ*M and 33 *μ*M, but converge into single stable states at the nutrient of 1000 *μ*M. Consistent with our findings in Fig. 2, these results demonstrate dramatic nutrient modulations of the circuit-host system.

Importantly, the above results reveal not only the shift of the circuit’s phase diagram but also the steady-state characteristics of the host and its modulation by nutrient, which elucidates the intricate dependence between the circuit and the host and showcases the power of integrative modeling.

### Transient responses of the switch-host system upon nutrient shifts

**Figure 4:**
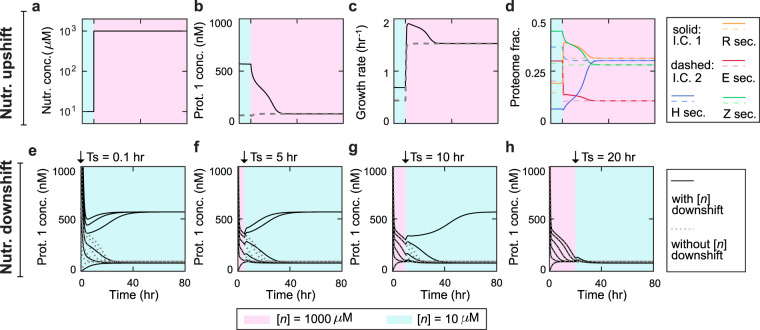
Transient responses of the circuit and the host upon nutrient shifts. (**a**) Profile of nutrient upshift from 10 to 1000 *μ*M at time 0. (**b**–**d**) Responses of Protein 1 (**b**), host growth (**c**) and proteome (**d**) upon the nutrient upshift. Solid and dashed lines correspond to the responses when the systems starts from the two steady states at nutrient of 10 *μ*M. (**e**–**h**) Transient responses of Protein 1 ([*P*_1_]) upon the nutrient downshift from 1000 to 10 *μ*M at 0.1 (**e**), 5 (**f**), 10 (**g**), and 20 (**h**) hr. Solid lines are [*P*_1_] responses upon nutrient downshifts; dotted lines are [*P*_1_] responses when no downshift occurs.

Our steady-state results showed that both the circuit stability and host physiology can be influenced by nutrient conditions. This inspired us to examine the transient properties of the circuit-host system upon nutrient shifts because, in nature, nutrient level varies constantly. A unique feature of our integrative model is the ability to quantitatively capture the dynamic behaviors of both circuit and host^22^. Thus, we utilized the framework to determine how the circuit and the host respond transiently when the environment is switched between rich and poor nutrient conditions. We first performed an *in silico* nutrient upshift. Specifically, at *t* < 0, the system was settled in either of the two steady states (solid and dashed lines) at a low nutrient level (10 *μ*M); then, at *t* = 0, the nutrient was switched to a high level (1000 *μ*M) (Fig. 4a). The experiment showed that the upshift causes [*P*_1_] of the circuit, which is originally either in the stable low (dashed lines) or high (solid lines) states, to adapt to a single new steady state after a brief transient (Fig. 4b). This transition is due to the fact that, at a nutrient level of 10 *μ*M, the system is bistable and both the circuit and host variables possesses two stable states but at 1000 *μ*M the system becomes monostable. Accordingly, the growth rate, initially in one of the two stable states, converges to a single rate (Fig. 4c) with the increase of nutrient level. The cellular proteome also transits from two stable partitions to a single, new partition as shown in Fig. 4d.

We then tested the transient responses of the circuit-host system upon a nutrient downshift from 1000 to 10 *μ*M, which corresponds to the alteration of the system from monostability to bistability. In principle, in a bistability regime a system in different initial states may evolve to different steady states. We thus speculated that, depending on the downshift time, the system can exhibit distinct transient patterns as well as steady-state behaviors. To test this speculation, we conducted *in silico* nutrient downshifts at different time points after the initiation of temporal evolution (Fig. 4e–h). Indeed, when the nutrient downshift occurs early (e.g., the transition time *T*_*s*_ < 0.1 hr), [*P*_1_] evolves to two distinct stable states for different initial conditions (Fig. 4e). With the increase of the shifting time (5 hr and 10 hr), fewer trajectories lead to the high [*P*_1_] state (Fig. 4f–g). Eventually, when the shift occurs after a sufficiently long time (*T*_*s*_ > 20 hr), the system converges into the same steady state regardless of its initial states (Fig. 4h). The results demonstrated that a fully dynamic, mechanistic model is capable of elucidating transient circuit and host dynamics for nutrient shifts. The results also showed that the shifting time can be critical to the final state of the switch-host system.

### Steady-state switch and host behaviors under varied antibiotic stresses

Like nutrient availability, environmental stresses often vary in natural settings and have prominent impacts on cell physiology. The integrative model incorporates key cellular processes such as translation, which allows us to mechanistically describe cellular stresses such as the effects of chloramphenicol, a ribosome-inactivating antibiotic^30^. We thus used the framework to explore how chloramphenicol concentration ([*C*_*m*_]) affects the steady-state switch and host behaviors in a rich nutrient (1000 *μ*M) environment.

**Figure 5:**
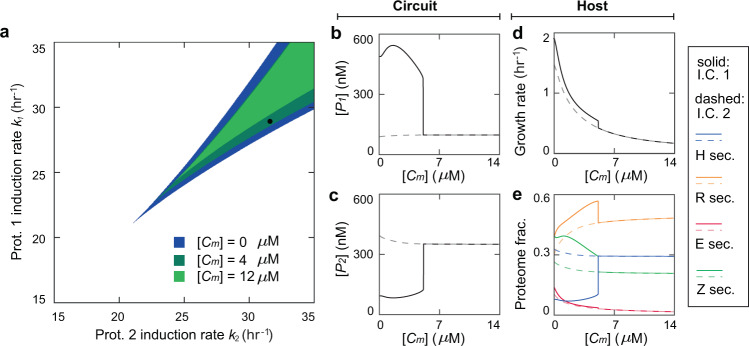
Steady-state circuit and host behaviors under chloramphenicol stress. (**a**) Bistability diagrams at various levels of chloramphenicol concentration [*C*_*m*_]. The colored areas are bistability regions; the blue, dark green and green colors correspond to the chloramphenicol of 0, 4 and 12 *μ*M accordingly. The black dot (*k*_1_ = 29 *hr*^−1^, *k*_2_ = 31.6 *hr*^−1^) is the specific condition under which the circuit-host system is further explored in Panel b–e. (**b**,**c**) Steady-state concentrations of Protein 1 ([*P*_1_]) (**b**) and Protein 2 ([*P*_2_]) (**c**) as a function of [*C*_*m*_]. The protein level is bistable when [*C*_*m*_] is below 4.2 *μ*M and monostable otherwise. (**d**) Steady-state growth rate of the host as a function of [*C*_*m*_]. The growth exhibits a similar bistatility-to-monostability transition as the circuit proteins. (**e**) Proteome mass fractions as functions of [*C*_*m*_]. Blue, orange, red and green lines correspond to heterologous circuit proteins (H sector), ribosomal proteins (R section), enzyme proteins (E section) and remaining proteins (Z section) respectively. For Panel b–e, solid and dash lines correspond to the steady states evolved from I.C. 1 and I.C. 2 respectively.

Figure 5a shows the bistability phase space of the circuit for varied chloramphenicol levels (0 (dark blue), 4 (dark green) and 12 (green) *μ*M). Here, the bistability region for [*C*_*m*_] = 0 *μ*M is identical to the case of the nutrient level 1000 *μ*M in Fig. 2a. These results show that the bistability region reduces with increasing [*C*_*m*_], which can be understood as follows: the percentage of deactivated ribosomes increases with enhancing [*C*_*m*_], which effectively reduces the overall translational capacity of the cell. To remain bistable, the toggle switch requires higher induction levels (*k*_1_ and *k*_2_) to overcome the reduction in translational capacity. Consequently, the circuit transitions from bistability to monostability with increasing [*C*_*m*_] for a given set of *k*_1_ and *k*_2_ such as the dot in Fig. 5a (*k*_1_ = 29 hr^−1^ and *k*_2_ = 31.6 hr^−1^). Additionally, the results show that the lower edge of the phase boundary is affected differently from the upper edge, which is due to the differential loads of the two circuit proteins.

Along with the bistability analysis, we also examined the responses of circuit and host variables to chloramphenicol variations. Figure 5b,c shows steady-state circuit protein behaviors for a given set of induction strengths, corresponding to the dot in Fig. 5a, when [*C*_*m*_] is systematically altered. Clearly, the circuit proteins change from being bistable to monostable when [*C*_*m*_] is beyond a critical level (4.2 *μ*M). Interestingly, the maximal [*P*_1_] and the minimal [*P*_2_] from the initial condition 1 (solid lines in Fig. 5b,c) occur at a non-zero level of chloramphenicol ([*C*_*m*_] = 1.2 *μ*M), which arises from the balance between the reduction in translational capacity and reduced nutrient uptake. In concert with circuit protein profiles, the steady-state growth rate displays a transition from bistability to monostability (Fig. 5d) as is the case for nutrient shift (Fig. 2). However, the difference is that the overall growth rate increases with nutrient but declines monotonically with chloramphenicol due to the differential roles of the two environmental parameters. Similarly to the growth rate, the steady-state proteome allocations among the four coarse-grained sectors are modulated (Fig. 5e).

**Figure 6:**
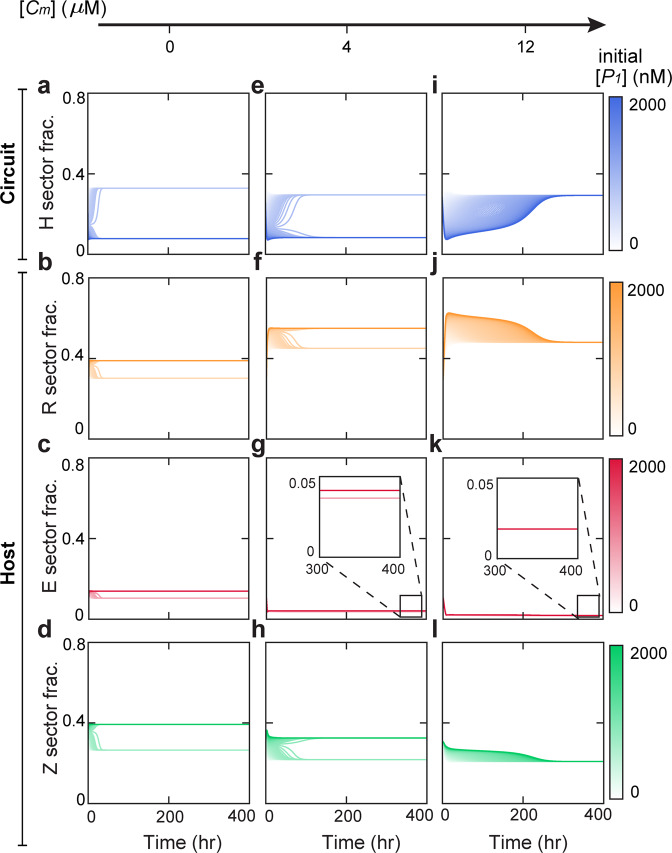
Temporal behaviors of cellular proteomes at different levels of chloramphenicol concentration ([*C*_*m*_]). The left, middle and right columns correspond to the chloramphenicol of 0, 4 and 12 *μ*M accordingly. For each panel, the colors of trajectories from light to dark correspond to altered initial Protein 1 concentration ([*P*_1_]) from 0 to 2000 nM; other initial conditions remain invariant. Parameters used here correspond to the dot in Fig. 5a.

We further simulated the temporal dynamics of circuit and host variables at different initial settings where the initial [*P*_1_] is altered from 0 to 2000 nM while others remain constant (Fig. 6 and Fig. S2). Consistent with our findings in Fig. 5, both the circuit (H sector) and the host parts (R, E, and Z sectors) of the proteome show bistability at low [*C*_*m*_] (the left and middle columns) but become monostable at high [*C*_*m*_] (the right column).

### Dynamic responses of the switch and the host to antibiotic shifts

**Figure 7:**
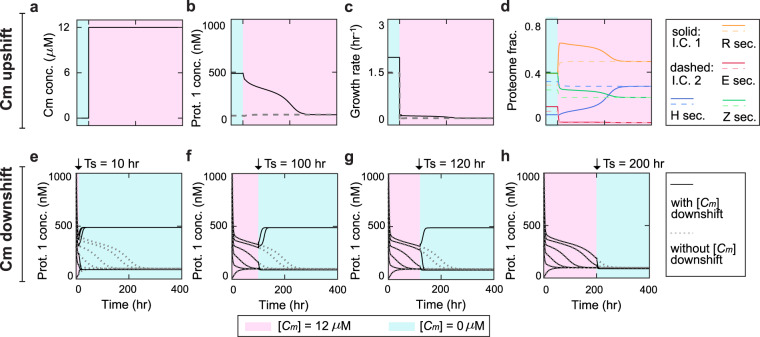
Transient responses of the circuit and the host upon chloramphenicol concentration ([*C*_*m*_]) shifts. (**a**) Profile of [*C*_*m*_] upshift from 0 to 12 *μ*M at time 0. (**b**–**d**) Responses of Protein 1 ([*P*_1_]) (**b**), host growth (**c**) and proteome (**d**) upon the upshift. Solid and dashed lines correspond to the system starting from the steady states evolved from I.C.1 and I.C. 2 accordingly. (**e**–**h**) Transient responses of Protein 1 ([*P*_1_]) upon chloramphenicol downshift at 10 (**e**), 100 (**f**), 120 (**g**) and 200 (**h**) hr. Solid lines are Protein 1 responses upon chloramphenicol downshifts; dotted lines are Protein 1 responses when no downshift occurs.

We also investigated the transient dynamics of the switch-host system upon antibiotic alterations. Similar to the case of nutrient shifts, the circuit-host system was first allowed to settle into one of the two steady states in the absence of chloramphenicol (i.e., [*C*_*m*_] = 0 *μ*M). Then, chloramphenicol level was shifted from 0 to 12 *μ*M (Fig. 7a) at time 0 and, subsequently, transient circuit behaviors and the host physiology were examined (Fig. 7b–d). Because the system is bistable at [*C*_*m*_] = 0 *μ*M but monostable at [*C*_*m*_] = 12 *μ*M, the upshift of the antibiotic leads to the convergence of [*P*_1_]. The system, initially in either of the two steady states, develops into a single steady state after a long transient time (Fig. 7b). Accordingly, the host variables, including both the growth rate (Fig. 7c) and proteome partition (Fig. 7d), experience the similar stability change. We also tested how chloramphenicol relaxation modulates the switch and host dynamics. Figure 7e–h shows the transient behaviors of the circuit’s Protein 1 when chloramphenicol is reduced from 12 to 0 *μ*M at different time points. Because the system is monostable at [*C*_*m*_] = 12 *μ*M but bistable at [*C*_*m*_] = 0 *μ*M, Protein 1 can evolve to different steady states, depending on differential initial conditions at time 0, when the downshift occurs relatively early (Fig. 7e–g). However, when the transition occurs late, Protein 1 converges to a single steady state regardless of its initial condition (Fig. 7h).

## Discussion

After successful creation of a wide array of engineered gene circuits, synthetic biology is now advancing into a new era wherein circuits are deployed in the real world. To aid in this transition, mathematical models are needed to quantitatively understand genetic devices in natural environments which fluctuate constantly. Our study shows that an integrative approach has the potential to mathematically describe environmental dependence of circuit-host dynamics. Specifically, the framework provides a mechanistic scheme to account for both the steady-state characteristics and transient responses of the circuit-host system upon nutrient and antibiotic variations. Our results also illustrate the complexity of gene circuit behavior in changing environments through intimate circuit-host coordination.

There are few remarks for our study. First, the proteomic fraction dedicated to heterologous proteins can be high. One key feature of our framework is being coarse-grained, which means proteins with similar biological functions or similar dynamic patterns can be grouped into a single effective protein as a way to simplify representation. Following this spirit, the proteins *P*_1_ and *P*_2_ here represent not only the specific transcriptional factors in the toggle switch (i.e., CI and LacI respectively)^7^ but also heterologous proteins whose productions are controlled by the switch. As the switch can be used to drive a number of genes with desired functions such as complex biosynthetic pathways^31^, the proteins encoded by the switch genes and the genes driven by the switch can be coarse-grained into effective *P*_1_ and *P*_2_. As a result, heterologous proteins can take up a large sector of the total proteome. To support the argument, a recent experiment showed that proteins over-expressed from a single gene (*lacZ*) can constitute about 30% of the total proteome^25^. Second, our results seem different from conclusions from a different recent coarse-grained approach^32^. Extended from the two-variable ODE representation in the original switch model^7^, the coarse-grained model in that study includes circuit-host competition by revising the production terms of the two proteins. However, it does not involve a detailed mechanistic description of host variables or resource allocation dynamics like our framework does. Additionally, the form of the revised production terms suggests that the other model primarily considers the competition of machineries such as RNAP and ribosomes; in contrast, our model includes the competition of machineries, energy and building blocks. Because of fundamental differences of model assumptions, the corresponding results may not be directly translatable in a strict one-to-one way with ours. One more key difference is that, in our study, we explore circuit dynamics under varied nutrient and antibiotic levels instead of metabolic load, because the focus of our paper is to investigate how environmental factors shape circuit dynamics. Third, while our study focuses on the behaviors of the toggle switch in *E. coli* only, one may be curious whether the results apply to different circuits and cellular hosts. For different synthetic circuits in the same host, we speculate that system behaviors can be described by revising the detailed circuit kinetics and recalibrating the circuit-host coupling while keeping intact for the host physiology. By contrast, for the same circuit in different organisms, the circuit module can remain unchanged but the description of the host physiology and circuit-host coupling need to be updated. Generally, we speculate that the degree of model revision correlates positively with the phylogenetic relevance between *E. coli* and the host of interest. As supported by our previous work^22^, our original model was successfully adapted to describe key physiological variables of *Salmonella typhimurium*, a species of proteobacteria closely related to *E. coli*, without any alterations to the model parameters. For comparison, to capture the more distantly-related, Gram-positive *Streptomyces coeliocolour*, the value of one key parameter needed to be revised while keeping the model structure and other parameters invariant.

As an initial exploration, this work demonstrates the promise of integrative modeling for understanding circuit behaviors in simple varying environments. Moving forward, it is valuable to examine circuit dynamics in more complex settings, such as environments with periodic or random external fluctuations^33^, intracellular conditions where biomolecules fluctuate intrinsically^34^ and habitats where multiple species of microbes coexist and their interactions vary^12,35–37^. More importantly, experiments need to be performed to test model predictions and, through experiment-modeling iterations, to achieve a quantitative understanding of circuit dynamics in complex environments.

## Methods

### Construction of a circuit-host model for the genetic toggle switch

To model circuit dynamics in varying environments, we employed an integrative circuit-host modeling framework we recently developed^22^. Different from traditional models focusing on circuit alone, the framework involves three fundamental parts: a coarse-grained description of host physiology, a detailed module for exogenous circuit kinetics and multiple layers of circuit-host coupling. The framework possesses a mechanistic description of host physiology, which is intrinsically subject to environmental variations, and an explicit consideration of circuit-host coupling which organically links circuit dynamics to host behaviors. Thus, the model is uniquely suited to model gene circuits in naturally fluctuating environments. For this study, the host part can be captured using a 12-variable system of ordinary differential equations (ODEs) focusing on amino acid flux. The circuit part can be modeled using four ODEs corresponding to the kinetics of the mRNAs and proteins of the two genes of the switch. Notably, one of the circuit proteins (Protein 2, *P*_2_) is assumed to consume more resources than the other (Protein 1, *P*_1_), as the case of experimental study^7^. Additionally, for simplicity, the two proteins are assumed functionally neutral. We primarily considered variations of nutrient and chloramphenicol levels in environments, because they are two key parameters regulating environments and essential to cellular growth and metabolism. A mathematical model of 16 variables was constructed to study the toggle switch and its host, which is detailed in Supplementary Information (Section 1). The ODEs were numerically simulated in MATLAB (MathWorks) using the ode15s solver with a relative and an absolute tolerance of 10^−6^.

### Parameters and initial conditions

Detailed information relating to the parameters, simulation methods and initial conditions of the study is provided in Supplementary Information (Section 2 and 3).

## Supplementary information

Supplementary Information.
