# Supplementary material for: Integrative Circuit-Host Modeling of a Genetic Switch in Varying Environments
Source: Sci Rep. 2020 May 20;10:8383. doi: 10.1038/s41598-020-64921-5 (PMC7239927; doi:10.1038/s41598-020-64921-5)
Supplement: Supplementary file 1 — Supplementary Information. [file 41598_2020_64921_MOESM1_ESM.pdf]

Supplementary Information for Integrative circuit-host modeling of a genetic toggle switch in varying environments

Jordan Sickle, Congjian Ni
Center for Biophysics and Quantitative Biology and Center for Advanced Bioenergy and Bioproducts Innovation
University of Illinois at Urbana-Champaign, Urbana, IL 61801, U.S.A.

Daniel Shen
Dougherty Valley High School, San Ramon, CA 94582, U.S.A.

Zewei Wang
Monte Vista High School, Danville, CA 94506, U.S.A.

Matthew Jin
University Laboratory High School, Urbana, IL 61801, U.S.A.

Ting Lu
Department of Bioengineering, Department of Physics, Center for Biophysics and Quantitative Biology,
Center for Advanced Bioenergy and Bioproducts Innovation and Institute for Genomic Biology Urbana
University of Illinois at Urbana-Champaign, Urbana, IL 61801, U.S.A.

Contents

1 Construction of the Integrative Switch Model **2**

2 Specific Initial Conditions I.C. 1 and I.C. 2 **5**

3 Simulation Methods, Parameters and Initial Conditions **6**

4 Supplementary Tables **8**

5 Supplementary Figures **13**

1 Construction of the Integrative Switch Model

The integrative switch model is based on the original framework [1], which combines a dynamic coarse-grained and mechanistic description of host physiology with a synthetic toggle switch through bidirectional coupling. The model can be described in terms of host and circuit parts.

Host part

The host proteome is divided into three coarse-grained sectors based on their functions in the cell [2], namely ribosomal and affiliated proteins (r), transport and precursor synthesis enzymes (e), and remaining proteins (z). The framework describes the kinetics of the protein sectors (P_j, $j = r, e, z$) , their respective mRNAs (M_j, $j = r, e, z$), building blocks (amino acids A_a and ATP A_e), tRNA (t), rRNA (r_o) and ribosomes (R_o), as well as the global alamone ppGpp (s) [3–6]. All molecular species are subject to dilution caused by cellular growth (λ). The corresponding equations are given below

$$\frac{\mathrm{d}[s]}{\mathrm{d}t} = J_s^{in}(\cdot) - J_s^{out}(\cdot) - \lambda(\cdot)[s] \tag{S1}$$
$$\frac{\mathrm{d}[A_e]}{\mathrm{d}t} = J_e^{in}(\cdot) - J_e^{out}(\cdot) - (d_e + \lambda(\cdot))[A_e] \tag{S2}$$
$$\frac{\mathrm{d}[A_a]}{\mathrm{d}t} = J_a^{in}(\cdot) - J_a^{out}(\cdot) - (d_a + \lambda(\cdot))[A_a] \tag{S3}$$
$$\frac{\mathrm{d}[t_c]}{\mathrm{d}t} = J_{tc}^{in}(\cdot) - J_{tc}^{out}(\cdot) - \lambda(\cdot)[t_c] \tag{S4}$$
$$\frac{\mathrm{d}[r_o]}{\mathrm{d}t} = \gamma_{rr}(\cdot) - \lambda(\cdot)[r_o] \tag{S5}$$
$$\frac{\mathrm{d}[M_j]}{\mathrm{d}t} = \gamma_j(\cdot) - (\pi_{0,j} + \lambda(\cdot))[M_j] \qquad j = r, e, z \tag{S6}$$
$$\frac{\mathrm{d}[P_j]}{\mathrm{d}t} = \beta_j(\cdot) - \lambda(\cdot)[P_j] \qquad j = r, e, z \tag{S7}$$
$$\frac{\mathrm{d}[R_o]}{\mathrm{d}t} = k_{ro}^{+}([r_o] - [R_o])([P_r] - [R_o]) - k_{ro}^{-}[R_o] - \lambda(\cdot)[R_o] \tag{S8}$$

where J_x^{in} represents the synthesis of component x and J_x^{out} describes the degradation and/or consumption of x.

Circuit part

The circuit part contains detailed kinetics for the mRNAs and proteins of the genes within a circuit. In this study, the circuit is a two-gene toggle switch; thus, a total of four equations are introduced below

$$\frac{\mathrm{d}[M_1]}{\mathrm{d}t} = \gamma_{P1}(\cdot) - (\pi_{0,P1} + \lambda(\cdot))[M_1] \tag{S9}$$
$$\frac{\mathrm{d}[P_1]}{\mathrm{d}t} = \beta_{P1}(\cdot) - \lambda(\cdot)[P_1] \tag{S10}$$
$$\frac{\mathrm{d}[M_2]}{\mathrm{d}t} = \gamma_{P2}(\cdot) - (\pi_{0,P2} + \lambda(\cdot))[M_2] \tag{S11}$$
$$\frac{\mathrm{d}[P_2]}{\mathrm{d}t} = \beta_{P2}(\cdot) - \lambda(\cdot)[P_2] \tag{S12}$$

where $[M_1]$ and $[P_1]$ are the concentrations of mRNA and protein of gene 1, and $[M_2]$ and $[P_2]$ are the concentrations of mRNA and protein of gene 2.

Detailed expression

Detailed expression for the Eqs. (S1)-(S12) are provided below.

$$J_s^{in} = k_{s,0} + k_s[R_{st}] \tag{S13}$$

$$J_s^{out} = d_s[s] \tag{S14}$$

$$[R_{st}] = \sum_{j=r,e,z} \left(\frac{l_j \kappa_j^{ini} K_c[t_u]}{\kappa_j^{elo} K_u[t_c]} \right) \left(1 + \frac{K_{e,tl}}{[A_e]} \right) \left(\frac{[R_f]}{W_{r,j} + [R_f]} [M_j] \right) \tag{S15}$$

$$J_e^{in} = \frac{k_e[P_e][n]}{K_n + [n]} \cdot \frac{I_e}{I_e + [A_e]} \cdot \frac{[A_e]}{M_{e,e} + [A_e]} \tag{S16}$$

$$J_e^{out} = q_a J_a^{in} + q_{tc} J_{tc}^{in} + q_r \sum_{j=rr,r,e,z,\mathbf{h}} n_j \gamma_j + q_p \sum_{j=r,e,z,\mathbf{h}} l_j \beta_j + q_o \lambda(\cdot) \tag{S17}$$

$$J_a^{in} = \frac{k_a[P_e][n]}{K_n + [n]} \cdot \frac{I_a}{I_a + [A_a]} \cdot \frac{[A_e]}{M_{e,a} + [A_e]} \tag{S18}$$

$$J_a^{out} = J_{tc}^{in} \tag{S19}$$

$$J_{tc}^{in} = \frac{k_{tc}[P_r]}{1 + \frac{D_a}{[A_a]} + \frac{D_e}{[A_e]} + \frac{D_u}{[t_o] - [t_c]}} \tag{S20}$$

$$J_{tc}^{out} = \sum_{j=r,e,z,\mathbf{h}} l_j \beta_j \tag{S21}$$

$$[t_o] = f_t[r_o] \tag{S22}$$

$$\beta_j = \frac{\kappa_j^{ini}[R_f]}{W_{r,j} + [R_f]}[M_j] \qquad j = r, e, z, P1, P2 \tag{S23}$$

$$[R_o] = [R_f] + \sum_{j=r,e,z,P1,P2} \left[\frac{1}{\kappa_j^{ini}} + \frac{l_j}{\kappa_{j,\text{eff}}^{elo}(\cdot)} + \frac{1}{\kappa_j^{ter}} \right] \beta_j(\cdot) \tag{S24}$$

$$\gamma_j = \frac{g_j(\cdot)}{V(\cdot)} \cdot \frac{\nu_j^{ini}[X_f]}{W_{x,j} + [X_f]} \cdot \alpha_{s,j}([s]) \qquad j = rr, r, e, z \tag{S25}$$

$$\gamma_{P1} = \frac{g_{P1}(\cdot)}{V(\cdot)} \cdot \frac{k_1[X_f]}{W_{x,P1} + [X_f]} \cdot \alpha_{s,P1}([s]) \cdot \frac{K_{i,P2}^{\theta_{P2}}}{K_{i,P2}^{\theta_{P2}} + [P_2]^{\theta_{P2}}} \tag{S26}$$

$$\gamma_{P2} = \frac{g_{P2}(\cdot)}{V(\cdot)} \cdot \frac{k_2[X_f]}{W_{x,P2} + [X_f]} \cdot \alpha_{s,P2}([s]) \cdot \frac{K_{i,P1}^{\theta_{P1}}}{K_{i,P1}^{\theta_{P1}} + [P_1]^{\theta_{P1}}} \tag{S27}$$

$$[X_o] = [X_f] + \frac{[X_f]}{W_{x,ns} + [X_f]} \cdot \frac{g_{ns}}{V(\cdot)} + \sum_{j=rr,r,e,z,P1,P2} \left[\frac{1}{\nu_j^{ini} \alpha_{s,j}(\cdot)} + \frac{n_j}{\nu_{j,eff}^{elo}(\cdot)} + \frac{1}{\nu_j^{ter}} \right] \gamma_j(\cdot) \tag{S28}$$

$$[X_o] = m_o[R_o] \tag{S29}$$

$$g_{rr} = \sum_{k=1}^{7} \exp(\lambda[(1 - x_{rr,k})C + D]) \tag{S30}$$

$$g_r = \frac{1}{19}\sum_{k=1}^{19} \exp(\lambda[(1 - x_{r,k})C + D]) \tag{S31}$$

$$g_e = \frac{1 - e^{-\lambda C}}{1 - e^{-\lambda C/m_e}} e^{\lambda(C+D)} \tag{S32}$$

$$g_z = \frac{1 - e^{-\lambda C}}{1 - e^{-\lambda C/m_z}} e^{\lambda(C+D)} \tag{S33}$$

$$g_{P1} = g_{0,P1}\frac{\exp(\lambda(C + D)) - \exp(\lambda D)}{\lambda C} \tag{S34}$$

$$g_{P2} = g_{0,P2}\frac{\exp(\lambda(C + D)) - \exp(\lambda D)}{\lambda C} \tag{S35}$$

$$\alpha_{s,j}([s]) = \frac{\epsilon_{s,j} + \xi_{s,j}([s]/W_{s,j})^{\theta_{s,j}}}{1 + ([s]/W_{s,j})^{\theta_{s,j}}} \qquad j = rr, r, e, z, P1, P2 \tag{S36}$$

$$\kappa^{elo}_{j,eff} = \kappa^{elo}_{j} \frac{1}{1 + \dfrac{K_c}{[t_c]} + \dfrac{K_c}{[t_c]} \cdot \dfrac{[t_u]}{K_u} + \left(\dfrac{[C_m]}{K_t}\right)^{\theta_t}} \cdot \frac{[A_e]}{K_{e,tl} + [A_e]} \qquad j = r, e, z, P1, P2 \tag{S37}$$

$$\nu^{elo}_{j,eff} = 3\kappa^{elo}_{j,eff} \qquad j = r, e, z, P_1, P_2 \tag{S38}$$

$$\nu^{elo}_{rr,eff} = \nu^{elo}_{rr}\frac{[A_e]}{K_{nt} + [A_e]} \cdot \frac{[A_e]}{K_{e,tr} + [A_e]} \tag{S39}$$

$$\lambda = \frac{1}{\rho}\sum_{j=r,e,z,P1,P2} l_j\beta_j \tag{S40}$$

$$V = V_0 \exp\left(\frac{c_v[n]^{\theta_n u}}{W_\nu^{\theta_\nu} + [n]^{\theta_\nu}}\right) \tag{S41}$$

Notably, all variables and parameters are listed in Tables S1, S2, and S3.

Specific to our study, two environmental variables, nutrient level (n) and chloramphenicol concentration (C_m), are explicitly expressed in the model. Here, nutrient serves as the substrate for amino acid and ATP productions (Eqs. (S16), (S18)) and determines cell volume (Eq. (S41)); chloramphenicol decreases the translation rate by inhibiting translation elongation (Eq. (S37)).

Additionally, the mass fractions for the proteome r, e, z and h sectors (f_r, f_e, f_z and f_h) are calculated as

$$f_r = \frac{l_r[P_r]}{\sum\limits_{j\in\{r,e,z,P_1,P_2\}} l_j[P_j]} \tag{S42}$$

$$f_e = \frac{l_e[P_e]}{\sum\limits_{j\in\{r,e,z,P_1,P_2\}} l_j[P_j]} \tag{S43}$$

$$f_z = \frac{l_z[P_z]}{\sum\limits_{j\in\{r,e,z,P_1,P_2\}} l_j[P_j]} \tag{S44}$$

$$f_h = \frac{l_{P1}[P_1] + l_{P2}[P_2]}{\sum\limits_{j\in\{r,e,z,P_1,P_2\}} l_j[P_j]} \tag{S45}$$

2 Specific Initial Conditions I.C. 1 and I.C. 2

To determine the circuit's multistability (e.g. phase diagrams in Figs. 2 and 5), we compare the system's steady states from the simulations starting with two initial conditions: the initial condition 1 (I.C. 1) and the initial condition 2 (I.C. 2). I.C. 1 corresponds to a high level of Protein 1 ($[P_1]$), a high level of mRNA 1 ($[M_1]$), a low level of P_2 ($[P_2]$), and a low level of mRNA 2 ($[M_2]$); I.C. 2 corresponds to a low level of Protein 1 ($[P_1]$), a low level of mRNA 1 ($[M_1]$), a high level of P_2 ($[P_2]$), and a high level of mRNA 2 ($[M_2]$). Given the mutual inhibition of the two switch genes, simulating the system with these two initial conditions enables us to determine system multistability. The system is defined bistable if the steady states from the two initial conditions do not converge and monostable otherwise.

To utilize the above approach, one key requirement is to ensure the initial low levels of biomolecules (i.e., protein and mRNA) to be sufficiently low and high levels to be sufficiently high. Thus, for the low levels, we choose zeros as the initial conditions, i.e.,

$$[M_j]_{low} = 0 \tag{S46}$$

$$[P_j]_{low} = 0 \qquad j = 1, 2 \tag{S47}$$

For the high levels, we use the theoretical upper bounds of protein and mRNA levels. Specifically, by setting the Eqs. (S9)-(S12) to be zeros, we have the steady states of the circuit ($[M_j]_{ss}$ and $[P_j]_{ss}$) and their upper bounds as

$$\begin{aligned}[M_j]_{ss} &= \frac{g_j(\cdot)}{V(\cdot)} \cdot \frac{\nu_j^{ini}[X_f]}{W_{x,j} + [X_f]} \cdot \alpha_{s,j}([s]) \cdot \frac{K_{i,j'}^{\theta_{j'}}}{K_{i,j'}^{\theta_{j'}} + [P_{j'}]^{\theta_{j'}}} \frac{1}{\pi_{0,j} + \lambda(\cdot)} \\ &< \frac{g_j(\cdot)}{V(\cdot)} \cdot \frac{\nu_j^{ini}}{\pi_{0,j}} < \frac{\nu_j^{ini}}{CV_0\pi_{0,j}} \cdot \frac{g_{0,j}\exp(\lambda(C+D))}{\lambda}\end{aligned} \tag{S48}$$

$$[P_j]_{ss} = \frac{\kappa_j^{ini}[R_f]}{W_{r,j} + [R_f]} \frac{[M_j]_{ss}}{\lambda(\cdot)} < \kappa_j^{ini} \frac{[M_j]_{ss}}{\lambda(\cdot)} < \frac{\kappa_j^{ini}\nu_j^{ini}}{CV_0\pi_{0,j}} \cdot \frac{g_{0,j}\exp(\lambda(C+D))}{\lambda^2} \tag{S49}$$

from which we determine our initial high mRNA and protein levels as

$$[M_j]_{high} = \frac{g_{0,j} M_{g,coeff} \max(k_1, k_2)}{CV_0\pi_{0,j}} = 3.98 \times 10^5 \text{ nM} \tag{S50}$$

$$[P_j]_{high} = \frac{g_{0,j} \kappa_j P_{g,coeff} \max(k_1, k_2)}{CV_0\pi_{0,j}} = 1.53 \times 10^8 \text{ nM} \qquad j = P1, P2 \tag{S51}$$

where we use the parameters in Tables S2 and S3 along with $\nu_j^{ini} \leq \max(k_1, k_2)$ and

$$M_{g,coeff} = \max\left(\frac{\exp(\lambda(C+D))}{\lambda}\right) \tag{S52}$$

$$P_{g,coeff} = \max\left(\frac{\exp(\lambda(C+D))}{\lambda^2}\right) \tag{S53}$$

Thus, I.C. 1 has $[M_1] = 3.98 \times 10^5$ nM, $[P_1] = 1.53 \times 10^8$ nM, $[M_2] = 0$ nM, and $[P_2] = 0$ nM. In contrast, I.C. 2 has $[M_1] = 0$ nM, $[P_1] = 0$ nM, $[M_2] = 3.98 \times 10^5$ nM, and $[P_2] = 1.53 \times 10^8$ nM.

The initial conditions for the host variables are less sensitive to system steady-state behaviors. As detailed in the next section, two sets of initial host variable values are used to reduce the simulation cost.

Additionally, we consider the system in a steady state if the relative reaction rate is less than 10^{-6}, i.e.,

$$\frac{|[\dot{P_1}]|}{[P_1]} < 10^{-6} \text{ and } \frac{|[\dot{P_2}]|}{[P_2]} < 10^{-6} \tag{S54}$$

3 Simulation Methods, Parameters and Initial Conditions

The model (Eqs. (S1)-(S12)) is solved numerically in time with our custom MATLAB code involving the ODE solver *ode15s*. The transcription rates γ_j from Eqs. (S25)-(S29) and translation rates β_j from Eqs. (S23)-(S24) are solved algebraically using non-linear equation solver *lsqnonlin*.

All parameter values, except k_1 and k_2, are listed in Tables S2 and S3. The former contains parameters adopted from the previous model [1] and the latter contains those introduced in this study. In Table S3, the copy numbers of Protein 1 and Protein 2 genes, $g_{0,P1}$ and $g_{0,P2}$, are both set to be one. The lengths of Protein 1 (l_{P1}) and Protein 2 (l_{P2}) are 5×10^3 and 1×10^6 amino acids respectively, to reflect different metabolic loads. Here, the amino acids include not only those of the proteins from the switch genes but also those by the genes regulated by the switch. Thus, the amino acid lengths can be large in principle. The maximum transcription initiation rates, k_1 and k_2, are varied between 15 hr^{-1} and 35 hr^{-1}.

In Fig. 2a, the phase diagrams are generated by varying both k_1 and k_2 in the range from 15 hr^{-1} to 35 hr^{-1}, with a resolution of 800 $\times$ 800 pixels, for three nutrient levels (10, 33 and 1000 μM). In Fig. 2b-e, the steady states are plotted as functions of nutrient for the induction strength set (k_1, k_2) = (25, 28) hr^{-1}. The I.C. 1 and I.C. 2 are used for both Fig. 2a and Fig. 2b-e.

In Fig. 3, the time courses of proteome fractions are illustrated for the induction strength set (k_1, k_2) = (25, 28) hr^{-1}. Initial host variables are fixed, the circuit mRNAs are set to be 0 nM, and Protein 2 is fixed at 500 nM. The initial value of Protein 1 ranges from 0 to 2000 nM.

In Fig. 4a-d, nutrient upshifts are simulated from the steady states of the system at the induction strength set (k_1, k_2) = (25, 28) hr^{-1}. In Fig. 4e-h, nutrient downshifts are simulated from the initial conditions where both mRNAs are set to be 0, Protein 2 is fixed at 1000 nM but Protein 1 is varied from 0, 600, 1200, 1800, 2400, to 3000 nM.

In Fig. 5a, the phase diagrams are generated by varying both k_1 and k_2 in the range from 15 hr^{-1} to 35 hr^{-1} for three chloramphenicol levels (0, 4 and 12 μM). In Fig. 5b-e, the steady states are plotted as functions of nutrient for the induction strength set k_1 = 29 hr^{-1} and k_2 = 31.6 hr^{-1}. The I.C. 1 and I.C. 2 are used for both Fig. 5a and Fig. 5b-e.

In Fig. 6, the time courses of proteome fractions are illustrated for the induction strength set (k_1, k_2) = (29, 31.6) hr^{-1}. The same initial conditions as in Fig. 3 are used.

In Fig. 7a-d, chloramphenicol upshifts are simulated from the steady states of the system at the induction strength set (k_1, k_2) = (29, 31.6) hr^{-1}. In Fig. 7e-h, nutrient downshifts are simulated from the initial conditions as those in Fig. 4e-h are used.

Our simulations in the study also involve initial conditions for the host variables. In Fig. 2 and Fig. 5, the initial conditions of the host variables for both I.C. 1 and I.C. 2 are $[s] = 302\,\mu M$, $[A_e] = 179\,\mu M$, $[A_a] = 0.74\,\mu M$, $[t_c] = 0.32\,\mu M$, $[r_o] = 35.2\,\mu M$, $[M_r] = 9.2\times10^{-4}\,\mu M$, $[P_r] = 25.9\,\mu M$, $[M_e] = 0.126\,\mu M$, $[P_e] = 3771\,\mu M$, $[M_z] = 0.14\,\mu M$, $[P_z] = 4270\,\mu M$ and $[R_o] = 25.8\,\mu M$. In Fig. 3, 4e-h, 6, 7e-h, the initial conditions of host variables are $[s] = 21.23\,\mu M$, $[A_e] = 1646.91\,\mu M$, $[A_a] = 1047.33\,\mu M$, $[t_c] = 357.20\,\mu M$, $[r_o] = 90.22\,\mu M$, $[M_r] = 0.32\,\mu M$, $[P_r] = 91.92\,\mu M$, $[M_e] =$

$2.52\,\mu M$, $[P_e] = 1499.80\,\mu M$, $[M_z] = 7.28\,\mu M$, $[P_z] = 4325.46\,\mu M$, and $[R_o] = 89.23\,\mu M$. In Fig. 4a-d, Fig. 7a-d, the simulations started from the corresponding steady states.

4 Supplementary Tables

Table S1: Description of variables

symbol	description
s	ppGpp
A_e	ATP
A_a	amino acid
t_c	charged tRNA
t_u	uncharged tRNA
r_o	rRNA
M_j	mRNA associated with gene j
P_j	protein associated with gene j
R_o	total ribosome
R_f	free ribosome
X_o	total RNA polymerase
X_f	free RNA polymerase
g_j	gene copy number for gene j
$\alpha_{s,j}$	regulation factor of ppGpp on gene j
β_j	protein production rate for protein j
$\kappa_{j,eff}^{elo}$	effective translation elongation rate for protein j
γ_j	transcription rate for gene j
$\nu_{j,eff}^{elo}$	effective transcription elongation rate for mRNA j
λ	cell growth rate
V	cell volume
n	nutrient
Cm	chloramphenicol

Table S2: Description parameters and their values from the previous model [1].

symbol	value	description
C	6.70E-01 h	C period
D	3.30E-01 h	D period
g_{ns}	4.60E+06	number of non-specific genomic binding sites
$W_{x,ns}$	3.10E+03 μM	Michaelis constant
ν_{rr}^{ini}	6.60E+03 hr^{-1}	maximum transcription initiation rate
$W_{s,rr}$	4.00E+01 μM	dissociation constant of ppGpp regulation
$\theta_{s,rr}$	2.00E+00	Hill coefficient of ppGpp regulation
ν_{rr}^{elo}	3.06E+05 nucl. hr^{-1}	maximum chain elongation rate
$W_{x,rr}$	3.00E-01 μM	dissociation constant of RNA polymerase binding
n_{rr}	6623	number of nucleotides in a rRNA gene
K_{ntp}	5.00E+01	dissciation constant of nucleotide addition for rRNA synthesis
$x_{rr,1}$	5.00E-02	location of rRNA gene 1
$x_{rr,2}$	1.16E-01	location of rRNA gene 2
$x_{rr,3}$	1.00E-02	location of rRNA gene 3
$x_{rr,4}$	2.38E-01	location of rRNA gene 4
$x_{rr,5}$	1.30E-01	location of rRNA gene 5
$x_{rr,6}$	5.58E-01	location of rRNA gene 6
$x_{rr,7}$	4.22E-01	location of rRNA gene 7
l_r	13624	number of amino acids in a R-sector gene
ν_r^{ini}	1.50E+03 hr^{-1}	maximum transcription initation rate
$W_{s,r}$	4.00E+01 μM	dissociation constant of ppGpp regulation
$\theta_{s,r}$	1.00E+00	Hill coefficient of ppGpp regulation
$W_{x,r}$	7.00E-01 μM	dissociation constant of RNA polymerase binding
$\pi_{0,r}$	8.32E+00 hr^{-1}	mRNA degradation rates
κ_r^{ini}	2.04E+03 hr^{-1}	maximum translation initiation rate
$W_{r,r}$	1.30E+00 μM	dissociation constant of ribosome binding
$W_{r,r,pr}$	1.00E+00 μM	dissociation constant of r-protein mRNA self-binding
κ_r^{elo}	9.00E+04 a.a. hr^{-1}	maximum translation chain elongation rate
$x_{r,1}$	1.95E-01	location of r-protein gene 1
$x_{r,2}$	1.92E-01	location of r-protein gene 2
$x_{r,3}$	1.97E-01	location of r-protein gene 3
$x_{r,4}$	1.83E-01	location of r-protein gene 4
$x_{r,5}$	1.20E-01	location of r-protein gene 5
$x_{r,6}$	1.21E-01	location of r-protein gene 6
$x_{r,7}$	2.53E-01	location of r-protein gene 7
$x_{r,8}$	9.05E-01	location of r-protein gene 8
$x_{r,9}$	3.29E-01	location of r-protein gene 9
$x_{r,10}$	7.34E-01	location of r-protein gene 10
$x_{r,11}$	4.97E-01	location of r-protein gene 11
$x_{r,12}$	4.02E-01	location of r-protein gene 12
$x_{r,13}$	2.27E-01	location of r-protein gene 13
$x_{r,14}$	2.97E-01	location of r-protein gene 14

$x_{r,15}$	2.25E-01	location of r-protein gene 15
$x_{r,16}$	6.97E-01	location of r-protein gene 16
$x_{r,17}$	3.78E-02	location of r-protein gene 17
$x_{r,18}$	8.14E-01	location of r-protein gene 18
$x_{r,19}$	6.40E-03	location of r-protein gene 19
m_e	300	number of E-sector genes
l_e	300	number of amino acids in a metabolic protein
ν_e^{ini}	9.00E+01 hr^{-1}	maximum transcription initiation rate
$W_{s,e}$	4.00E+01 μM	dissociation constant of ppGpp binding
$\theta_{s,e}$	1.00E+00	Hill coefficient of ppGpp binding
$W_{x,e}$	7.00E-01 μM	dissociation constant of RNA polymerase binding
$\pi_{0,e}$	8.32E+00 hr^{-1}	mRNA degradation rate
κ_e^{ini}	2.04E+03 hr^{-1}	maximum translation initiation rate
$W_{r,e}$	1.30E+00 μM	dissociation constant of ribosome binding
κ_e^{elo}	9.00E+04 a.a. hr^{-1}	maximum translation elongation rate
m_z	300	number of Z-sector genes
l_z	300	number of amino acids in a Z-sector protein
ν_z^{ini}	9.00E+01 hr^{-1}	maximum transcription initiation rate
$W_{x,z}$	7.00E-01 μM	dissociation constant of RNAp binding
$\pi_{0,z}$	8.32E+00 hr^{-1}	mRNA degradation rate
κ_z^{ini}	2.04E+03 hr^{-1}	maximum translation initiation rate
$W_{r,z}$	1.30E+00 μM	dissociation constant of ribosome binding
κ_z^{elo}	9.00E+04 a.a. hr^{-1}	maximum translation elongation rate
$W_{s,P1}$	6.00E+01 μM	dissociation constant of ppGpp binding
$\theta_{s,P1}$	2.00E+00	Hill coefficient of ppGpp binding
$\pi_{0,P1}$	8.85E+00 hr^{-1}	mRNA degradation rate
κ_{P1}^{ini}	2.50E+02 hr^{-1}	maximum translation initiation rate
κ_{P1}^{elo}	9.00E+04 aa hr^{-1}	maximum translation elongation rate
$W_{d,P1}$	1.00 μM	dissociation constant
θ_{d_P1}	1.00	Hill coefficient
$W_{x,P1}$	7.00E-01 μM	dissociation constant
ϵ_{P1}	1.00	basal level of ppGpp activation
ξ_{P1}	1.00	induced level of ppGpp activation
$K_{i,P1}$	2.00E-01 μM	Hill constant of Protein 2 inhibition on Protein 1 transcription
$W_{r,P1}$	8.00E-01 μM	dissociation constant of ribosome binding
$W_{s,P2}$	6.00E+01 μM	dissociation constant of ppGpp binding
$\theta_{s,P2}$	2.00E+00	Hill coefficient of ppGpp binding
$\pi_{0,P2}$	8.85E+00 hr^{-1}	mRNA degradation rate
κ_{P2}^{ini}	2.50E+02 hr^{-1}	maximum translation initiation rate
κ_{P2}^{elo}	9.00E+04 a.a. hr^{-1}	maximum translation elongation rate
$W_{d,P2}$	1.00 μM	dissociation constant
θ_{d_P2}	1.00	Hill coefficient

$W_{x,P2}$	7.00E-01 μM	dissociation constant
ϵ_{P2}	1.00	basal level of ppGpp activation
ξ_{P2}	1.00	induced level of ppGpp activation
$W_{r,P2}$	8.00E-01 μM	dissociation constant of ribosome binding
$K_{i,P2}$	2.00E-01 μM	Hill constant of Protein 1 inhibition on Protein 2 transcription
$K_{e,tl}$	2.70E+01 μM	ATP dependence of peptide elongation rate
K_c	5.50E+01 μM	dissociation constant of charged-tRNA binding
K_u	2.00E+02 μM	dissociation constant of uncharged-tRNA binding
K_t	1.00E+00 μM	dissociation constant of antibiotics
θ_t	1.00E+00	Hill coefficient
m_o	1.67E-01	coefficient of proportionality
ρ	3.00E+06 μM	total protein concentration in E. coli cells
f_t	4.00E+00	coefficient of proportionality
k_a	7.20E+03 μM hr^{-1}	maximum amino acid biosynthesis rate
K_n	7.00E+01 μM	Michaelis constant of nutrient importation
k_{tc}	1.44E+05 μM hr^{-1}	tRNA aminoacylation rate
D_a	4.00E+02 μM	dissociation constant of amino acid binding
D_u	8.00E+00 μM	dissociation constant of uncharged tRNA binding
D_e	3.00E+02 μM	dissociation constant of ATP binding
k_e	5.40E+04 μM hr^{-1}	maximum ATP biosynthesis rate
I_a	2.00E+03 μM	dissociation constant of amino acid feedback inhibition
I_e	4.00E+03 μM	dissociation constant of ATP feedback inhibition
M_{ea}	1.23E+02 μM	Michaelis constant of ATP dependence for amino acid production
M_{ee}	1.00E+00 μM	Michaelis constant of ATP dependence for ATP production
d_{aa}	5.00E-03 hr^{-1}	amino acid utilization rate by other non-specified pathways
d_{atp}	1.00E-01 hr^{-1}	ATP utilization rate by other non-specified pathways
q_a	2	number of ATP consumed per amino acid synthesized
q_r	10	number of ATP consumed per mRNA nucleotide bond formation
q_p	2	number of ATP consumed per peptide bond formation
q_{tc}	2	number of ATP consumed per tRNA aminoacylation
q_o	1.00E+06 μM	ATP cost in growth-related processes
$k_{0,s}$	3.60E+00 μM hr^{-1}	basal level of ppGpp synthesis
k_s	3.60E+03 μM hr^{-1}	induced level of ppGpp synthesis
d_s	1.26E+02 hr^{-1}	ppGpp degradation rate
$k_{rib,+}$	9.00E+02 (μMh)$^{-1}$	forward rate of rRNA and r-protein binding
$k_{rib,-}$	2.48E+01 hr^{-1}	reverse rate of rRNA and r-protein binding
V_0	2.06E-01 μ^3	cell volume at birth
c_v	1.98E+00	coefficient of proportionality
θ_v	1.23E+00	Hill coefficient
W_v	1.00E+01 μM	dissociation constant
$P_{g,coeff}$	2.36E+01	maximum value of $e^{\lambda(C+D)}/\lambda^2$
$M_{g,coeff}$	1.54E+01	maximum value of $e^{\lambda(C+D)}/\lambda$

Table S3: Description of parameters values assigned in the current study

symbol	value	description
$g_{0,P1}$	1.00E+00	copy number of Protein 1 gene
k_1	varied	maximal transcription initiation rate
l_{P1}	5.00E+03	number of amino acids in Protein 1
$g_{0,P2}$	1.00E+00	copy number of Protein 2 gene
k_2	varied	maximal transcription initiation rate
l_{P2}	2.50E+06	number of amino acids in Protein 2

5 Supplementary Figures

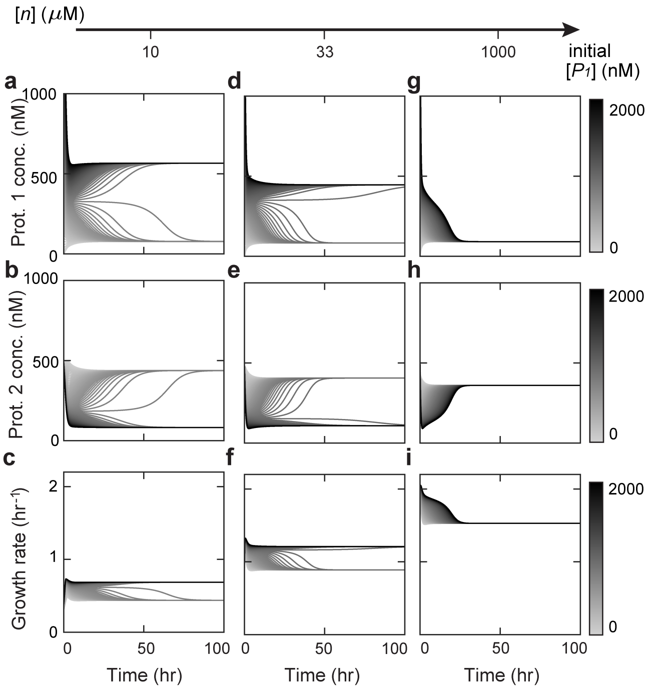

Figure S1: Temporal dynamics of protein 1, protein 2 and growth at different nutrient levels ($[n]$). The left, middle and right columns correspond to the nutrient of 10, 33, and 1000 μM accordingly. For each panel, the colors of the trajectories from light to dark correspond to altered the initial concentration of Protein 1 ($[P_1]$) from 0 to 2000 nM. Other initial conditions remain invariant.

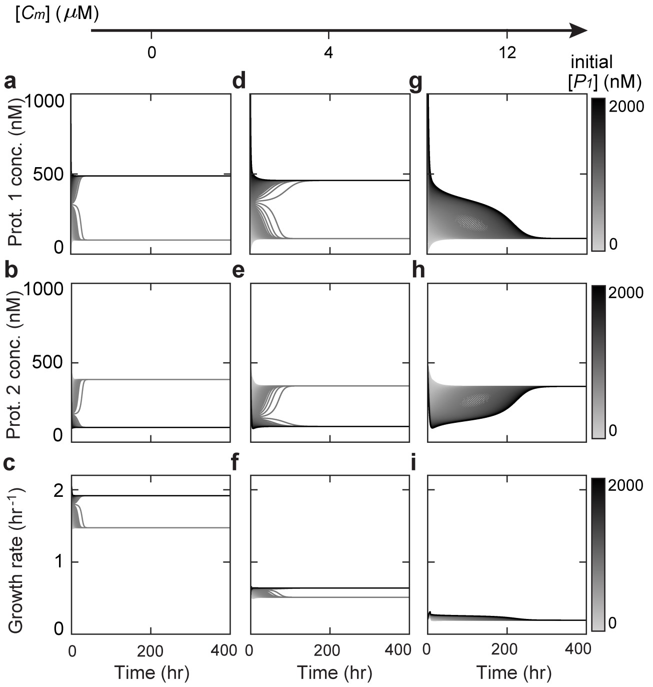

Figure S2: Temporal behaviors of protein 1, protein 2 and grwoth at different levels of chloramphenicol concentration ($[C_m]$). The left, middle and right columns correspond to the chloramphenicol of 0, 4 and 12 μM accordingly. For each panel, the colors of trajectories from light to dark correspond to altered initial Protein 1 concentration ($[P_1]$) from 0 to 2000 nM; other initial conditions remain invariant.

References

[1] Chen Liao, Andrew E. Blanchard, and Ting Lu. An integrative circuithost modelling framework for predicting synthetic gene network behaviours. *Nat. Microbiol.*, 2(12):1658–1666, Dec 2017.

[2] Matthew Scott, Carl W. Gunderson, Eduard M. Mateescu, Zhongge Zhang, and Terence Hwa. Interdependence of cell growth and gene expression: origins and consequences. *Science*, 330(6007):1099–1102, Nov 2010.

[3] Katarzyna Potrykus, Helen Murphy, Nadège Philippe, and Michael Cashel. ppGpp is the major source of growth rate control in E. coli. *Environ. Microbiol.*, 13(3):563–575, Mar 2011.

[4] Matthew F. Traxler, Sean M. Summers, Huyen-Tran Nguyen, Vineetha M. Zacharia, G. Aaron Hightower, Joel T. Smith, and Tyrrell Conway. The global, ppGpp-mediated stringent response to amino acid starvation in Escherichia coli. *Mol. Microbiol.*, 68(5):1128–1148, Jun 2008.

[5] Zachary D. Dalebroux and Michele S. Swanson. ppGpp: magic beyond RNA polymerase. *Nat. Rev. Microbiol.*, 10(3):203–212, Mar 2012.

[6] Patrick P. Dennis, Mans Ehrenberg, and Hans Bremer. Control of rRNA synthesis in Escherichia coli: a systems biology approach. *Microbiol. Mol. Biol. Rev.*, 68(4):639–668, Dec 2004.
